# What patients want to know about genetic testing for kidney disease

**DOI:** 10.3389/fmed.2023.1201712

**Published:** 2023-06-05

**Authors:** Judy Savige, B. André Weinstock

**Affiliations:** ^1^Department of Medicine (Melbourne Health and Northern Health), Royal Melbourne Hospital, The University of Melbourne, Parkville, VIC, Australia; ^2^Alport Syndrome Foundation, Scottsdale, AZ, United States

**Keywords:** genetic kidney disease, Alport syndrome, steroid-resistant nephrotic syndrome, patients, cystic kidney disease, tubulopathies, complementopathies

## Abstract

Previously, genetic kidney disease was often recognised when family members shared clinical features. Now, many genetic kidney diseases are diagnosed when testing demonstrates a pathogenic variant in a gene associated with the disease. Detection of a genetic variant also identifies the mode of inheritance, and suggests family members at risk. The genetic diagnosis has additional advantages for patients and their doctors even when no specific treatment is available since it often indicates likely complications in other organs, the clinical course, and management strategies. Generally, informed consent is required for genetic testing because the result provides “certainty” with implications for the patient, and their family, and possibly for employment, and for life and medical insurance, as well as having social, ethical, and financial consequences. Patients want to be provided with a copy of their genetic test result in a format that is comprehensible and to have the result explained. Their at-risk family members should be sought out and offered genetic testing too. Patients who allow the sharing of their anonymised results in registries help advance everyone’s understanding of these diseases and expedite a diagnosis in other families. Patient Support Groups not only help normalise the disease but also educate patients, and update them on recent advances and new treatments. Some registries encourage patients to themselves submit their genetic variants, clinical features and response to treatment. More and more often, patients may volunteer for clinical trials of novel therapies including some that depend on a genetic diagnosis or variant type.

## Background

Genetic kidney disease is common. The commonest monogenic kidney disease is AD Alport syndrome which affects one in 100 of the population (1) and causes haematuria but kidney failure much less often (2). The commonest genetic causes of kidney failure are AD polycystic kidney disease (ADPKD) which affects one in 1,000 (3) and X-linked Alport syndrome which occurs in one in 2,000 (1).

### When should a genetic kidney disease be suspected?

Previously a genetic disease was suspected because there was another affected family member, such as a parent, grandparent or sibling, with similar clinical features. However clinical features are not necessarily the same in affected family members, for example, one may have a single kidney while another has kidney cysts. Sometimes several family members have kidney failure but the cause is unknown. There are also many instances where there is only one affected family member, and the disease is still genetic. These sporadic cases occur because the families are small, disease inheritance is autosomal recessive (AR) or *de novo*, or features are incompletely penetrant. In such cases the clinician suspects a genetic disease for other reasons.

Thus some genetic diseases are suspected because the cause is usually genetic. Genetic kidney disease is a common cause of Congenital Abnormalities of the Kidney and Urinary Tract or CAKUT, where there are structural abnormalities with, for example, a single kidney or reflux nephropathy, and of Cystic kidney disease and the Renal Ciliopathies. Genetic kidney disease is also a common cause of some glomerulopathies including Alport syndrome and Focal and Segmental Glomerulosclerosis (FSGS), and of the tubulopathies such as Bartter and Gitelman syndrome.

Sometimes clinical features in these conditions overlap so that one family member may be diagnosed with Alport syndrome, and another with FSGS, cysts or kidney failure. Although all the affected family members have the same pathogenic variant, different features may predominate in different family members.

Another reason why genetic disease is suspected is that many genetic kidney diseases have features that affect other organs, in particular, the ears, eyes, heart or skeleton. It may be the ophthalmologist or audiologist who first recognises the diagnosis.

In addition, many genetic kidney diseases are diagnosed in children or younger people.

Finally, sometimes genetic kidney disease is suspected simply because there is no other obvious explanation.

More recently, with the increasing ubiquity of Massively Parallel Sequencing for paediatric screening, a genetic diagnosis may be made unexpectedly.

### What are the benefits of genetic testing?

Patients, family members and their clinicians may be eager for a diagnosis (Table 1). With the more widespread uptake and decreasing cost, genetic testing often provides the diagnosis as well as having other advantages (Table 2).

**Table 1:** What patients with a genetic kidney disease want.

To know their genetic diagnosis and what it means for them
For their treating clinicians to know their diagnosis and to optimise their management
To know their genetic variant and its significance
To minimise the harm for other family members
To bring some benefit to other people with the disease
To contribute to future research and help decide research priorities
To contribute to recommendations made in expert guidelines on disease management
To receive support from other patients, including support that “normalises” their disease
To belong to a Patient Support Group—for information, access to treatments, and to support research and activism
To access education about the disease, its causes and the risks for family members
To promote awareness of their disease
To help complete enrolment in clinical trials evaluating new treatments

**Table 2:** Patient expectations of genetic testing.

To provide an accurate diagnosis with a name for their disease
To minimise the number of clinic visits and diagnostic tests
To identify the inheritance pattern, and who else in their family is at risk of being affected
To identify associated clinical manifestations such as hearing loss or heart abnormalities
To share their pathogenic variant and anonymised clinical data to help understand more about the disease
To suggest effective treatments, and avoid those that are unhelpful or harmful
To ensure affected family members do not act as kidney donors
To help inform reproductive planning
To help explain better the cause of their disease, its clinical features and to develop treatments

Identifying a genetic diagnosis avoids the multiple clinic visits, tests and time that may be needed otherwise. Genetic testing is often physically less invasive, especially when DNA is collected from cheek brushings. In addition the genetic test indicates the name of the disease which enables patients and their doctors to know what they are dealing with and the likely clinical course. The diagnosis often suggests undetected extra-renal clinical features. Genetic testing indicates the inheritance pattern (autosomal dominant, autosomal recessive, X-linked, digenic, or mitochondrial). The mode of inheritance suggests who else in the family may be affected, who might undergo “cascade” testing and who should not act as a kidney donor (4, 5). It also indicates the likelihood of any offspring developing the disease which helps with reproductive planning. Genetic testing may also indicate whether the mutation is severe enough to result in kidney failure at a younger age (6).

The genetic test and diagnosis may also indicate those treatments that are effective and those that are unhelpful. Thus, steroids which are used commonly in immune-mediated FSGS are unhelpful in FSGS due to Alport syndrome. In these cases, the genetic diagnosis of Alport syndrome means that a child or adult avoids steroid-associated complications. Genetic testing also facilitates enrolment into clinical trials of new treatments. Even when a genetic kidney disease has no known treatment it is possible that minimising proteinuria or improving blood pressure control delays kidney failure (7–9). Conversely, genetic testing may exclude a diagnosis and expedite the use of more appropriate treatments.

A genetic diagnosis also unites people with a disease in Patient Support Groups.

### What are the risks of genetic testing for patients?

Before undertaking a genetic test, patients should consider the risks and benefits, and prepare themselves for the outcome. It may be helpful to have a discussion with a clinical geneticist or a genetic counsellor, around issues of consent, and the risk for other family members too.

For patients, genetic testing usually brings “certainty” of their diagnosis with consequences for themselves and their families. There are also implications for employment, and for life and medical insurance in different countries. Parents may feel guilty that they have passed on a disease to their children, or that it is unfair that they are unaffected but that another family member is. Indeed, even family members who have not been tested may find out that they too have a genetic kidney disease because of the inheritance pattern.

In addition, the results of genetic testing may be inconclusive. Currently only 60% of individuals with suspected Alport syndrome have a pathogenic variant demonstrated when Whole Exome Sequencing is used (10). The missing pathogenic variants may be copy number changes (insertions or deletions) which are detected less often with Whole Exome Sequencing, non-canonical splicing variants, or genetic variants that affect another gene but result in similar clinical features (11). Inconclusive genetic testing may also be due to a Variant of Uncertain Significance (“VUS”) where there is insufficient evidence to conclude that the change is Pathogenic or Likely Pathogenic.

On the other hand, genetic testing is not always available or may be too expensive when the individual is responsible for the cost.

Nevertheless there are also strategies to mitigate these risks (Table 3).

**Table 3:** Risks of genetic testing and mitigation strategies for the patient and clinician.

Risk	Mitigation strategy
Anxiety about the result	Preparing with the help of a genetic counsellor, considering the alternatives, and understanding that it is usually better to know their diagnosis because of possible treatments
Genetic testing is not available	Alternative tests such as a kidney biopsy or a biochemical test. However these may not be as accurate, as specific or as informative as genetic testing
Testing is too expensive	Some specialised research laboratories are able to test the patient without charge if they can use the anonymised information in their research, for example, to determine how common a disease is, or its natural history.
The result is inconclusive	Review at clinician request or by laboratory protocol at a later time.
No treatment is available	Things change, sometimes rapidly. Sometimes “general” treatments such as improving blood pressure control are helpful
Implications for employment and life insurance	Sometimes there is no way to avoid these issues. However for employment and insurance applications, significant family history must often be declared. Sometimes there is a government-mandated moratorium on genetic information being included in insurance assessments. Overall it is important to recognise that it is generally better to identify a diagnosis that will enable effective treatment
Other family members may learn that they are affected even though they have not been tested	This is sometimes unavoidable. However family members may have suspected the diagnosis anyway and may still benefit from more optimised management
Sadness because family members are affected	Realisation that inheritance and family members being affected are associated with the disease itself and that no one is to blame
“Guilt” at not themselves being affected	Family members are affected depending on the pattern of inheritance

### What are the financial, social, and ethical implications of genetic testing?

These vary in different jurisdictions and health systems. Currently the few economic analyses available indicate that genetic testing results in a reduction in the time spent and the cost of the “diagnostic odyssey” (12, 13).

Even where no specific treatment is available for a genetic disease there may be treatments that delay kidney failure. Thus, in Alport syndrome, closer monitoring and better medical care, including renin-angiotensin-aldosterone- (RAAS) blockade and possibly Sodium-glucose co-transporter2 inhibitors or flozins (14), delay the need for dialysis or a kidney transplant as well as reducing the ill-health and cardiac risks associated with impaired kidney function. In general, the costs of dialysis are so high that slowing kidney failure progression by even only a few years demonstrates a clear cost-benefit ratio for genetic testing.

There are generally strict rules about privacy that ensure a person’s genetic diagnosis remains confidential.

### How can a person with suspected genetic kidney disease arrange genetic testing for themselves or a family member?

In general, patients are referred by their family doctor to a kidney specialist or geneticist for assessment. A recent US survey found that all nephrologists had treated patients with a genetic kidney disease and all reported that learning more about genetic kidney disease was important for them (15).

At the clinic visit, the specialist conducts a formal evaluation, and may request a genetic test after obtaining written informed Consent. The Consent forms include information on the risks and benefits, and possibly further specific consents, such as whether the patient is happy for their results to be made available to family members; and for the pathogenic variant to be submitted to national and international databases that can be accessed by other laboratories to help with their own patients’ diagnoses.

It is usually easier and faster for family members who are also at risk of disease to be identified with genetic testing after the variant in one definitely-affected family member has already been assessed as Pathogenic or Likely Pathogenic.

### How can a person at risk of a genetic kidney disease access genetic testing at no cost?

There are alternatives if the local healthcare system does not provide genetic testing. Testing is often accessed through the person’s treating kidney doctor who will know if testing is available and worthwhile. Patient Support Groups are often aware of strategies. In some countries, pharmaceutical companies offer free testing. This enables them to learn more about the disease course or “natural history,” that is, how often people develop kidney failure, and at what age. Some major international research laboratories provide free genetic testing for the very rare diseases they study. These laboratories must have Institutional Ethics approval, and there may be an expectation that the patient is happy for the laboratory to use and publish their variant and “deidentified” clinical data.

### What are the alternatives if genetic testing is not available?

Other approaches may also be used to confirm the diagnosis where a genetic disease is suspected. This may involve a specialised blood or urine test, a kidney or muscle biopsy, or radiological imaging. However genetic testing usually represents the simplest, most accurate and least invasive method for diagnosis.

### What do the genetic results include and how do patients learn the results?

There is an increasing trend for patients to have access to their own test results. This is mandated in the US under the Health Insurance Portability and Accountability Act of 1996 (HIPAA). There will be fewer misunderstandings about the results (16) when patients are also provided with written confirmation of their disease, with its name, the gene affected and the pathogenic variant with an explanation. Some kidney disease patient registries request patients submit their own pathogenic variants and clinical data. Patients who refer themselves to participate in clinical trials may need to know their gene affected in their disease and sometimes the variant type.

### What does a genetic report look like?

Reports are fairly standard internationally and many features are mandated by national bodies. Payment from the government or medical insurers is usually contingent upon accreditation of the testing laboratory and following “best practice.”

The reports use gene names in upper case and in italics such as *PKD1* or *COL4A5* and tend to describe a “Pathogenic variant” rather than “mutation.” Reports may also refer to the Reference Sequence for the gene which is the coding DNA that is accepted internationally for the gene with standardised numbering that indicates the location of the variant and the variant type. The patient should be told if they have a “Pathogenic” or “Likely Pathogenic” variant or a Variant of Uncertain Significance (VUS). The reports use the same “ACMG” criteria (17) to assess all variants so that conclusions are consistent between laboratories worldwide. The report may also indicate how often a VUS should be reviewed, for example, twice more at 18 month intervals after the initial assessment.

### What are the important features of a genetic test result?

The test result usually indicates the genetic diagnosis, the gene affected, the mode of inheritance, how many pathogenic variants are detected in the affected gene, and whether the variant is a missense or other change (mainly large rearrangements, deletions, or nonsense or splice site variant).

There are subtle variations in effect for each of these features. Thus for a woman with a single or heterozygous pathogenic missense variant in the *COL4A3* gene: a single copy of this variant is consistent with the diagnosis of AD Alport syndrome; and two copies with AR Alport syndrome. A person with AR Alport syndrome with two copies of a missense variant often has milder disease than someone with two copies of a nonsense change.

### How can a person find out their risk of being affected with a genetic disease?

Whether a person is affected or not, can only be determined with certainty by genetic testing and, less often, with another specific test. The nephrologist, clinical geneticist or genetic counsellor can help explain the risk of other family members being affected and may help coordinate testing at-risk family members.

Genetic testing has the advantage that it indicates both the diagnosis and the mode of inheritance, and sometimes also the *risk* for family members from the inheritance pattern of a disease and their sex without themselves undergoing testing. Thus all the daughters of a man with an X-linked disease such as X-linked Alport syndrome inherit the pathogenic variant. However none of the man’s sons inherit the disease because they do not inherit the X chromosome.

Nevertheless the genetics are usually more complicated than this. To determine if a family member is affected their doctor needs to know the disease, the gene, and the mode of inheritance, all of which are usually only available from genetic testing. The kidney specialist, clinical geneticist or a genetic counsellor can then explain a family member’s risk of disease. The risk for each family member of being affected is “independent” of the results for previous family members. This means that even though a person from a family with an AD disease is affected, the risk for each of their sibling is still 50%.

It is important for people with a genetic disease to inform their family if they too may be affected, and there is evidence that this occurs more often when the person is encouraged to do so when initially told their results by their doctor (18).

### Do women generally have a milder form of genetic kidney disease?

Men and women generally have the same risk of inheriting a genetic kidney disease and developing end-stage kidney failure. However with X-linked inheritance, for diseases such as X-linked Alport syndrome, Fabry disease and Dent disease, women inherit a pathogenic variant twice as often as men but have more variable clinical features ranging from mild to severe. The danger of believing that all women with X-linked disease have milder disease is that they are not monitored as closely as required. In addition, women with X-linked diseases still have half their sons and half their daughters inheriting the pathogenic variant and at risk of the disease itself.

### How can patients find out more about their genetic kidney disease?

The easiest way is to ask members of their treating team but Patient Support Groups are another source of information (Table 4). Support group websites are often monitored for accuracy by members, and the information is usually up-to-date. Patient Support Groups are another way for patients to meet people with the same disease, and often the same questions and concerns. They often have access to international experts in the disease who are happy to answer highly specialised questions. Websites are available in different languages in different countries but there is often collaboration between these groups. These sites are also a way to find out about clinical trials of new therapies.

**Table 4:** Some Patient Support Group websites.

Alport syndrome
Alport Syndrome Foundation (ASF): https://www.alportsyndrome.org/
Alport UK: http://www.alportuk.org/
Fabry disease
NFDF: https://www.fabrydisease.org/
Polycystic kidney disease (PKD)
PKDInternational: https://pkdinternational.org/membership
Nephrogenic diabetes insipidus (NDI)
HDIF: https://ndif.org/
von Hippel-Lindau disease (VHL)
VHLA: https://www.vhl.org/
Global genes
https://resource-hub.globalgenes.org/kb/article/298-gene-based-diagnosis-101/
Proteinuria: https://nephcure.org/connect/virtual-support-groups/
Tuberous sclerosis: https://www.tscalliance.org/

### How can a person with a genetic kidney disease access new treatments?

New treatment trials are often introduced soon after treatments are demonstrated to be safe and prior to commercial availability. Patients participating in these trials have access to medications before they are proven to be effective and while their side effects are not fully known. It is a common practice for patients in clinical trials to be randomised to the new treatment or, alternatively, current “best practice” or to no treatment. This process of randomisation cannot be influenced, so that a doctor cannot guarantee that their patient will receive the new treatment. However it is also common that participants in such trials who are randomised to the alternative or placebo arm are provided, at the conclusion of the study, with a course of the effective treatment for at least an equivalent period of time as study participants.

Some of the “new” treatments have been “repurposed” where they were previously used for another condition but have now been trialed for genetic kidney disease. Repurposed medications are often relatively inexpensive and their side-effect profile is well-established. Patient Support Groups have regular updates on new treatment trials.

### How can a person with genetic kidney disease contribute to our understanding of their disease and help other people with the same condition?

People can agree to their genetic variants being shared with other members of their family to help with their diagnoses. They may also allow their pathogenic variants and deidentified clinical information to be shared with the international research community in a variant database or registry. The variant database allows laboratory scientists to confirm that a variant found in one of their patients is also disease-causing. Patient registries are used for studies of the disease such as correlating variant types with clinical features and outcomes.

Patients can also participate in clinical trials that evaluate new treatments. Patient Support Group meetings help with the education of patients and clinical staff, lobby for effective treatments, host educational and scientific meetings, and fundraise for education, research and patient care.

## Conclusion

Nephrologists should consider genetic testing to be a highly accurate and sensitive, cost-effective tool for the diagnosis of genetic kidney disease.

People with genetic kidney disease and their at-risk family members should be actively supported in their request for genetic testing and provided with genetic counselling. People with a genetic kidney disease should be given a copy of their test result and preferably a written explanation.

People with a genetic kidney disease should be encouraged to join their Patient Support Group for education, information on clinical trials and registries, and to help prioritise research and participate in the writing of expert guidelines.

## Data availability statement

The original contributions presented in the study are included in the article/supplementary material, further inquiries can be directed to the corresponding author.

## Author contributions

JS developed the answers to these questions in collaboration with the patient BAW and both have contributed to writing the manuscript. All authors contributed to the article and approved the submitted version.

## Conflict of interest

The authors declare that the research was conducted in the absence of any commercial or financial relationships that could be construed as a potential conflict of interest.

## Publisher’s note

All claims expressed in this article are solely those of the authors and do not necessarily represent those of their affiliated organizations, or those of the publisher, the editors and the reviewers. Any product that may be evaluated in this article, or claim that may be made by its manufacturer, is not guaranteed or endorsed by the publisher.
